# Are short‐read amplicons suitable for the prediction of microbiome functional potential? A critical perspective

**DOI:** 10.1002/imt2.38

**Published:** 2022-07-04

**Authors:** Vitor Heidrich, Lukas Beule

**Affiliations:** ^1^ Centro de Oncologia Molecular Hospital Sírio‐Libanês São Paulo Brazil; ^2^ Departamento de Bioquímica, Instituto de Química Universidade de São Paulo São Paulo Brazil; ^3^ Julius Kühn Institute (JKI)—Federal Research Centre for Cultivated Plants Institute for Ecological Chemistry, Plant Analysis and Stored Product Protection Berlin Germany

**Keywords:** compositional data, functional potential profiles, intragenomic marker gene variability, microbial functions, microbiome data, short‐read amplicon sequencing, taxonomic resolution

## Abstract

Taxonomic marker gene analysis allows uncovering taxonomic profiles of microbial communities at low cost, making it omnipresent in microbiome research. There is an ever‐expanding set of tools to extract further biological information from this kind of data. In this perspective, we enunciate several concerns regarding the biological validity of predicting functional potential from taxonomic profiles, especially when they are generated by short‐read sequencing. The taxonomic resolution of marker genes, intragenomic variability of marker genes, and the compositional nature of microbiome data are discussed. Combining actual measurements of microbiome functions with predicted functional potentials is proposed as a powerful approach to better understand microbiome functioning. In this context, the significance of predicted functional potentials for generating and testing hypotheses is highlighted. We argue that functions of microbiomes predicted from microbiome DNA read count data generated by short‐read amplicon sequencing should not serve as the only basis to draw biological inferences.

## Highlights

- Concerns regarding the biological validity of predicting functional potential from taxonomic profiles of microbiome data sets are enunciated.
- Taxonomic resolution of marker genes, intragenomic variability of marker genes, and the compositional nature of microbiome data are discussed.
- Combining measurements of actual functions and predicted functional potential profiles is a powerful approach to understanding microbial functioning.

## Graphical abstract

We raise concerns regarding the biological validity of predicting functional potential from microbiome taxonomic profiles generated by short‐read amplicon sequencing. We reason that predicted functional potential profiles can be improved by employing long‐read sequencing technologies, which in combination with independent measurements of actual functions can constitute a powerful approach to understanding microbiome functioning.

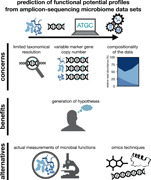

## INTRODUCTION

Over the last two decades, next‐generation sequencing (NGS) technologies gained enormous popularity for the analysis of microbial communities. Modern NGS platforms are affordable, allowing users to multiplex large amounts of samples, and have high base calling accuracy. These technologies enabled researchers to decipher the composition of microbial communities in varied habitats (e.g., [1, 2–3]). With the rapid rise in microbiome research, several tools emerged to predict functional potential profiles from the taxonomical profiles of microbiome data sets [4]. Functional potential profiles usually list the microbial functions predicted to be present in a microbiome as well as the relative importance of each function based on the proportion of microbes in which they are predicted to be present. As microbial functions are important for the basic understanding of microbial communities, these tools aim to complement taxonomical microbiome data sets. Despite all the additional information that such tools offer, we here communicate several concerns that we have regarding the use of taxonomic annotation of microbiome data sets generated by short‐read amplicon sequencing to derive microbial functions. While the prediction algorithms themselves have been discussed intensively (e.g., [4]), this perspective focuses on the applicability of short‐read amplicon sequencing data to the task of functional potential prediction, and not the efficiency of prediction algorithms.

### Taxonomic resolution of amplicon sequencing

At the time of writing, short‐read amplicon sequencing using the Illumina platform is the most common sequencing technology. On Illumina's widely used MiSeq System, short paired‐end reads of up to 2 × 300 base pairs can be generated. For the analysis of microbiomes, the most popular targets of short‐read amplicon sequencing are taxonomically informative parts of amplified 16S ribosomal RNA (rRNA) and internal transcribed spacer (ITS) loci. Sequencing of parts of these loci often lacks taxonomic resolution at species level. For example, almost two decades ago, Blackwood et al. [5] sequenced the hypervariable V1 to V3 regions of the 16S rRNA genes of *Bacillus* spp. and found that species of the clinically relevant *Bacillus cereus* group, *Bacillus* *anthracis* and *Bacillus cereus*, could not be distinguished. In fact, sequencing of full 16S rRNA genes of the *B*. *cereus* group revealed only some isolates of *B*. *anthracis* and *B*. *cereus* presented discriminable 16S rRNA sequences [6, 7], pointing to the limited taxonomic resolution of this marker gene for certain taxa. Recently, the importance of strain‐level identification of members of the human microbiome has been pointed out [8, 9]. Short‐read amplicon sequencing, however, mostly fails to differentiate closely related strains [9].

Previous studies proved the self‐evident fact that a full‐length 16S assessment, which is achievable by employing a long‐read sequencing technology such as PacBio, increases taxonomic resolution (e.g., [10]). As an alternative to long‐read sequencing, Loop Genomics proposed adapting Illumina short‐read sequencing technology to produce synthetic long reads by using unique molecular barcodes in each 16S amplicon, which also showed to improve taxonomic resolution at species level [11]. Additionally, by using computational tools such as SMURF [12], it is possible to infer taxonomic profiles with a greater taxonomic resolution by aggregating information regarding amplicon sequencing libraries targeting different 16S hypervariable regions. This alternative is currently more accessible because there is no need for replacing sequencing platforms or adapting library preparation workflows. However, this is still suboptimal since, in opposition to Loop Genomics technology, full‐length 16S sequences cannot be reconstructed with this strategy.

Although the costs are still prohibitive for wide adoption, we predict that full‐length 16S assessment either by long‐read amplicon sequencing or bioinformatics reconstruction of the full‐length 16S based on uniquely identified short reads will gradually replace short‐read amplicon sequencing in the long term, leading to increased taxonomic resolution and, thus, more accurate prediction of functional potential profiles.

Apart from taxonomic distinction, genomic variability among isolates of the same species further impedes the accurate prediction of functions. In 2004, Jaspers and Overmann [13] found that 11 isolates of *Brevundimonas alba* had identical full 16S rRNA gene sequences but high genomic diversity [13]. The authors were able to show that despite identical 16S rRNA gene sequences, these isolates showed pronounced differences in their physiology. This illustrates the need to isolate microorganisms to precisely assess their functions. Although the lack of reference genomes in public databases undoubtedly limits functional potential prediction tools [4], this example further illustrates that reference genome availability does not guarantee reliable functional potential inferences.

### Intragenomic variability of marker genes

Another complication in exploring microbiome structure using amplicon sequencing data is the variable copy number of marker genes in microbial genomes. Fungi can contain up to several hundred copies of rRNA genes (which are interspaced by ITS sequences), but this varies by order of magnitude across fungi [14]. Even among isolates of a single fungal species, copy numbers of 18S and 28S rRNA genes per genome can vary largely [14, 15, 16–17]. Lavrinienko et al. [18] speculate that nontranscribed regions such as ITS may have an even greater copy number variability. Similarly, as shown in Rainey et al. [19], bacteria may contain multiple copies of the 16S rRNA gene. Recent estimates indicate the median 16S rRNA gene copy number per bacterial species can vary between 1 and 19 [20]. Although tools to correct for the gene copy number variability of 16S rRNA genes in archaeal and bacterial genomes have been developed [21, 22], the reliability of gene copy number correction remains questionable [23, 24]. In addition, it is known for over two decades that the 16S rRNA genes in a single bacterial genome are not always identical (e.g., [25]). This may cause amplicon sequencing studies to identify 16S rRNA alleles within the same bacterial cell as pertaining to different species [26], which often leads to inflated diversity estimates [27]. Therefore, besides the obvious implications of variable marker gene copy numbers in attempts of estimating the relative contribution of each species in the environment under study (i.e., distortion of relative abundances), this variability sometimes translates into allele diversity, which can even confound microbiome membership. As functional potential profiles reverberate all biases in taxonomic profiles, intragenomic variability of marker genes confounds the relative importance estimation of potential functions and likely inflates the predicted diversity of functional potential profiles.

### The compositional nature of microbiome data

Because there is no relationship between the number of reads generated by NGS from a sample and the number of bacterial cells in that sample [28], bacterial reads do not translate into bacterial abundances. The number of reads generated for each taxon during NGS informs solely on the relative sizes of parts of the community, making NGS microbiome data sets compositional [29]. In other words, this means they unlock the relative sequencing read abundance of the taxa (i.e., proportions or frequencies) present in a microbial community, but because the size (microbial biomass) of the whole community remains unknown, they do not reveal absolute abundances of taxa [28–31]. Therefore, even if the predicted functional potential profile of a community matches the profile of its actually realized functional potential, the total population size of the community is still ignored which disables estimation of the magnitude of the functional potential. For example, given two compositionally identical microbiomes (microbiome A and B) that differ in their overall population size by factor two (microbiome A has double the population size of B), we would like to compare their predicted to their realized functional potential. Although their predicted functional potentials based on taxonomic profiles will be identical, microbiome A has double the realized functional potential due to the double population size. Great efforts have been undertaken to overcome this limitation of microbiome analysis through various ways of additional quantification of microorganisms (e.g., [28, 30, 32, 33, 34]); however, different quantification methods may introduce additional data variability [35].

A further strategy to circumvent the caveats of compositional data analysis is the use of ratios (division between elements of the composition, i.e., proportions) [36]. This is because the microbial load bias vanishes in ratios [37]. So if the microbiome function of interest can be expressed in terms of ratios between molecules/functions/processes, as is often the case in biological systems (e.g., carbon/nitrogen, albumin/globulin, neutrophil/lymphocyte), relative abundances of potential functions turn into valuable information and quantification of the microbial load becomes less critical. Although not always practical, such that our concerns regarding the compositional nature of the data still hold, we suggest the use of ratios whenever possible when analyzing functional potential profiles. Regardless, compositionally‐aware statistical methods, which employ ratio‐based transformations to make the data less contingent on community sizes, are also useful and should be favored in this setting [29].

Finally, although not discussed here, it is worth mentioning that PCR bias is a well‐known source of error that can distort community composition (e.g., [38]) and consequently predicted functional potential profiles.

### Measuring the actual function of the microbiome

Another but more demanding approach is to measure the actual functions of interest of the microbiome if possible. For example, processes regulated by environmental microbiomes such as enzymatic activity [39], greenhouse gas fluxes [40], and nitrogen fixation [41] can often be measured in situ. Likewise, the function can be assessed from human/animal gut microbiomes by orthogonal measures of stool metabolites, which has been successfully used to investigate the relationship between the gut microbiome and biotransformations that partly explain the interpatient variability in the efficacy and toxicity of several drugs [42], including immunosuppressants [43]. Measuring actual microbial functions is only an option if microbial communities are accessible to functional measurements, functionally active at the point of sampling, and have a sufficient population size and amount of sampling material to detect the functions. For microbiomes that do not meet these criteria, additional quantification of functional genes using techniques like real‐time PCR is a valuable addition to quantifying their genetic potential for a given function. It has to be noted though that these genetic potentials do not necessarily translate into microbial activities and processes [44, 45–46]. In this regard, omics techniques such as transcriptomics, proteomics, and metabolomics could assist with the exploration of expressed genetic potentials. We argue that a combination of the measurement of selected actual functions and the predicted functional potential profiles is a powerful approach to understanding microbial functioning.

### Generating and testing hypotheses

Despite their limitations, we recognize the particular potential of predicted functional potential profiles for the generation of novel hypotheses. However, it is important not to neglect that microbiomes are complex. Accordingly, functional potential profiles are high‐dimensional and can be hard to analyze. This means that oftentimes functional potential prediction tools lead to too many research directions so that generating straightforward hypotheses becomes difficult. We strongly encourage researchers to select meaningful hypotheses and to independently test these hypotheses whenever possible. For example, Zhang et al. [47] predicted the metabolic functions of gut microbiota in mice and successfully validated their predictions by using nuclear magnetic resonance‐based metabolomics. Likewise, Wu et al. [48] predicted altered biosynthesis pathways of the gut microbiome in patients with colorectal adenomas and colorectal cancer as compared to healthy individuals. The authors validated their predictions by quantifying genes from these pathways using real‐time PCR. Even though studies validating predicted functional potential profiles are the exception rather than the rule, these examples illustrate the power of predicted functional potential profiles to find and explore new research directions.

## SUMMARY

**Figure 1:**
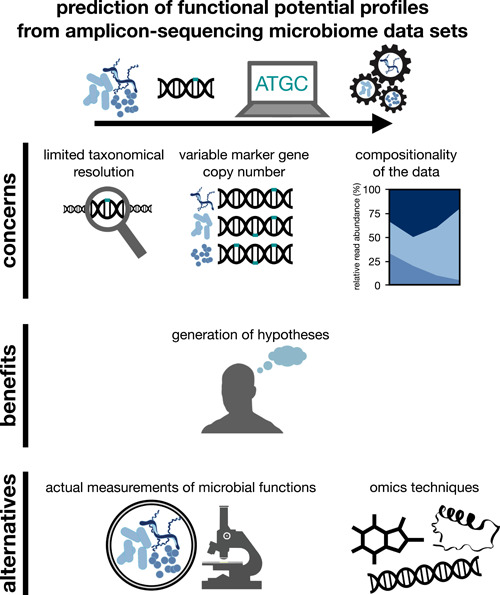
Schematic illustration of the concerns, benefits, and alternatives to the prediction of microbiome functional potential from amplicon sequencing microbiome data sets

An overview of the concerns, benefits, and alternatives to the prediction of microbiome functional potential discussed in this study is provided in Figure 1. Overall, we appreciate the efforts undertaken to enable the prediction of functional potential profiles from taxonomical microbiome data sets. We also believe that the prediction of functional potential profiles is useful to generate new ideas and explore new potential research directions. In conclusion, however, we argue that functions of microbiomes predicted from microbiome DNA read count data generated by short‐read amplicon sequencing should not serve as the only basis to draw biological inferences. We believe that the transition from short‐ to long‐read sequencing technologies will help to overcome some of the challenges presented here. Still, high‐resolution taxonomic profiling does not resolve crucial concerns we raised (e.g., the compositional nature of microbiome data). Therefore, measuring microbiome activity using omics (e.g., metabolomics) and non‐omics approaches (e.g., qPCR) will remain essential alongside taxonomic profiling to illuminate microbiome functioning.

## AUTHOR CONTRIBUTIONS

All authors listed have made a substantial, direct, and intellectual contribution to the work, and approved it for publication.

## CONFLICT OF INTEREST

The authors declare no conflict of interest.

## Data Availability

No new data and script were used in this study. Supplementary materials (figures, tables, scripts, graphical abstract, slides, videos, Chinese translated version, and updated materials) may be found in the online DOI or iMeta Science http://www.imeta.science
